# Molecular diagnosis appended by histopathological signature delineates the white spot syndrome virus (WSSV) infection in penaeid shrimps

**DOI:** 10.1016/j.cirep.2024.200138

**Published:** 2024-02-13

**Authors:** Md. Juwel Hasan, Shirin Sultana, Md. Nasir Khan, H.M. Rakibul Islam, Mohammad Nazrul Islam

**Affiliations:** a Department of Biotechnology, Sher-e-Bangla Agricultural University, Dhaka 1207, Bangladesh; b Fisheries Biotechnology Division, National Institute of Biotechnology, Savar, Dhaka 1349, Bangladesh; c Shrimp Research Institute, Bangladesh Fisheries Research Institute, Bagerhat 9300, Bangladesh

**Keywords:** Virus infection, Molecular detection, Prevalence rate, Histopathological studies, Shrimp diseases, Penaeus monodon

## Abstract

White spot disease (WSD) occurred by the highly devastating white spot syndrome virus (WSSV) poses a serious impediment toward global shrimp production. We investigated molecular diagnosis and natural infection with WSSV in cultured tiger shrimp (*Penaeus monodon*) and inspected histopathology of WSD. The incidence of WSSV infection was recorded in six small-scale shrimp farms situated in two districts of Bangladesh, namely Bagerhat and Satkhira. One- and two-step nested polymerase chain reaction models were applied to check the presence of WSSV DNA in shrimp samples. By one-step PCR, 25 of the 36 shrimp samples were WSSV-positive, while the remaining 11 samples were found to be WSSV-negative. After the negative samples underwent a two-step PCR process, only three samples were detected WSSV negative. In Satkhira and Bagerhat districts, the prevalence rates of WSSV infection were 83.33% and 55.56%, respectively, as confirmed by one-step PCR; following two-step nested PCR, the prevalence rates remained unchanged for Satkhira but increased to 100% for Bagerhat sourced samples. One-step PCR was less effective than two-step nested PCR in diagnosing viral infection; instantly evident that the overall prevalence rate rose from 69.44% to 91.67% when switching to two-step nested PCR. In a different experiment, histological analyses of PCR-confirmed WSSV-positive shrimp specimens revealed degenerated tubule lumen and degenerated hepatopancreas with necrosis, while the infected muscle tissues showed muscle fiber fragmentation and separation. Based on effective PCR technique and histopathological evidence of WSSV infection, the study demonstrated the occurrence of WSSV infection in small-scale shrimp farms. The combined practices of the diagnostic tools and information will be useful in understanding the white spot disease and managing its prevention in shrimp production systems.

## Highlights

• White spot syndrome virus infection is a grave concern in global shrimp production.
• Nested PCR detects increased prevalence of WSSV infection than one-step PCR alone.
• WSSV-positive shrimp shows necrotic and degenerated hepatopancreas & tubule lumens.
• WSSV infection causes fragmented muscle fibers with loss of myofibrils and striation.

## Introduction

Owing to the widespread presence and attack of various harmful virus and bacteria in shrimp production systems, the shrimp industries have to face a drastic less production [1]. According to an estimate, viral diseases caused a total of 60% of losses in the shrimp industry, while bacterial diseases were responsible for about 20% of losses [2]. The white spot syndrome virus (WSSV) is extremely virulent and the largest animal virus attacking the shrimps. On the contrary, shrimps are the crustacean invertebrates that lack effective adaptive immunity mechanism [3,4]. If the environmental stressors, stocking density and quarantine of broodstock and post-larva of shrimps are not well-controlled, it is very difficult to defend WSSV only with nonspecific innate immune system [3,5]. The cumulative mortality of a WSSV infection in cultured shrimp can reach 100% in just three to ten days after infection [6,7]. When WSSV first appeared in the southeast of Bangladesh in 1994, it spread to tiger shrimp (*Penaeus monodon*) cultured stocks in Cox's Bazar's semi-intensive farms, which led to a 50–60% reduction in shrimp production. Later, it has been exhibiting severe outbreaks also in small-scale farms in Bangladesh, causing serious economic loss every year. The WSSV infection caused white spot disease (WSD) in shrimp, resulting in an enormous annual loss to the world's economy [1]. It is reported that the rate of average loss will be projected to increase by $1 billion annually, which represent about 10% of the world's shrimp production [7].

Occurrence of numerous white spots throughout the exoskeleton, particularly on the carapace and abdomen of shrimps is one of the distinctive features of the WSSV infection. Even in the absence of WSSV infection, bacterial infections in the cuticle can be linked to the formation of white inclusions [8]. However, the identification of WSSV requires more than just the existence of white spots in the cuticle. Numerous methods are frequently employed to identify viral diseases, such as immunological approaches, conventional PCR [9], RT-PCR techniques [5], and so forth. When compared to one-step PCR, nested PCR offers a significant merit with a high degree of sensitivity [10]. Through single-step PCR, WSSV can be easily identified in shrimp when it manifests clinically in body tissue [11] but latent phase of viral infection or asymptomatic condition is efficiently identified by two-step or nested PCR [10]. But in the situations where both clinical symptoms and histological alterations are noted, histopathology has proven to be the gold standard for identifying WSSV. Sometimes, histopathological changes of the disease syndrome of *P. monodon* caused by different viral infection are very similar to each other; however, without exploring the viral DNA, it is not possible to identify appropriate causative agent. Along with the confirmation of WSD with clinical signs and PCR test, histopathology is a strong diagnostic tool [12]. Prevalence rate of WSSV infection was investigated in shrimps collected from commercial hatcheries in Bangladesh using PCR techniques by Ayub et al. [10] but histopathological experiment followed by PCR confirmation of WSSV infection was dim explored consecutively. In the present study, both two-step nested PCR and histopathological approaches were applied to diagnose and interpret the WSSV infection in penaeid cultured shrimps obtained from small-scale shrimp farms.

## Materials and methods

### Study area and shrimp sample collection

Shrimp samples were collected from local shrimp farms located in the southern regions of Bangladesh namely, Satkhira and Bagerhat districts during April to May 2021. From each district, three small scale farms (locally called *ghers*) owned by the marginal farmers were selected and 6–8 shrimp samples were randomly collected and placed individually within the zipped polybag on icebox. For histopathological investigation, four diseased and suspected shrimps were collected from currently affected shrimp farm in Bagerhat where symptoms of WSSV infection was tentatively confirmed through visual inspection and farmer experience. Two fresh and apparently healthy shrimp specimens were sampled from a rearing pond at Shrimp Research Institute (SRI), Bangladesh Fisheries Research Institute (BFRI), Bagerhat, where individual shrimp was maintained as healthy ones. Following collection, the samples were processed in the Shrimp Research Institute's laboratory using Davidson Fixative Solution. Finally all the samples were transported to and preserved in the Laboratory of Biotechnology, Sher-e-Bangla Agricultural University, Dhaka and Fisheries Biotechnology Division, National Institute of Biotechnology (NIB), Savar, Dhaka, Bangladesh.

### Genomic DNA isolation

Individual shrimp sample was aseptically taken out from the polybags using autoclaved forceps, and was placed on a blotting paper to blot off any excess water. Using autoclaved scissors, 20–30 mg of pleopod samples from each shrimp sample were cut into small pieces and transferred into microcentrifuge tubes. Whole genomic DNA was extracted by using commercial Tissue DNA Extraction Kit (Vivantis, Malaysia) followed by manufacturer protocol. Genomic DNA was quantified using a spectrophotometer and validated by electrophoresis on a 1% agarose gel.

### PCR primers and amplification

For the PCR test, a total of three primer pairs were utilized (Table 1). The sequence of a cloned 1461 bp WSSV DNA fragment was used to create the primer pairs 146F1/146R1 (outer primers) and 146F2/146R2 (inner primers), which amplify 1447 bp and 941 bp fragments of the WSSV DNA, respectively Lo et al. [13]. Since, the total genomic DNA was used as template, another primer pair, 143F/145R producing an 848 bp PCR product, originated from a highly conserved region of the decapod 18S rRNA sequence [13]. For the purpose of normalizing the PCR result and assessing the quality of the DNA extracted from shrimp samples, the primer pair 143F/145R was employed as an endogenous control.

**Table 1:** Primer sequences synthesized to amplify the white spot syndrome virus (WSSV) and shrimp DNA fragments (N represents G, A, T or C).

**Primer code**	**Primer sequence (5′–3′)**	**Product size (bp)**	**Origin**	**References**
146F1	ACTACTAACTTCAGCCTATCTAG	1447	WSSV specific outer primers	Lo *et al*. [13] and GenBank accession no. MN840357.1
146R1	TAATGCGGGTGTAATGTTCTTACGA			
146F2	GTAACTGCCCCTTCCATCTCCA	941	WSSV specific inner primers	
146R2	TACGGCAGCTGCTGCACCTTGT			
143F	TGCCTTATCAGCTNTCGATTGTAG	848	Decapod's conserved 18SrRNA sequence specific primers	Lo et al. [13]
145R	TTCAGNTTTGCAACCATACTTCCC			

#### One-step PCR

Both one-step PCR and nested PCR reactions were performed with two pairs of WSSV specific primers as described by Lo et al. [13] to detect the viral infection. To conduct a one step PCR, a 25 µl reaction mixture were prepared in a PCR tube by adding a 2X master mix containing GoTaq® DNA polymerase, dNTPs, MgCl_2_ and reaction buffer and adding forward and reverse primers (100 pmol each), DNA template (100 ng) and nuclease free water. A thermal cycler was used to perform the PCR amplification. The initial denaturation step took three minutes at 95 °C. Thereafter, there were 35 cycles of one minute denaturation at 95 °C, one minute annealing at 55 °C, one minute elongation or extension at 72 °C, and a final step of extension for five minutes at 72 °C.

#### Two-step or nested PCR

In a two-step nested PCR, the template DNA for the second step of PCR amplification was the amplicon from the first step. The outer primer pair, 146F1/146R1, was used for the first-step amplification which was carried out using specific thermal profile including an initial denaturation at 95 ºC for 3 min, followed by 15 cycles of 95 ºC for 1 min, annealing at 55 ºC for 1 min, elongation at 72 ºC for 1.5 min, and a final extension at 72ºC for 5 min. Once first-step PCR was finished, reaction mixtures were made using its amplicon (10 µl) as template and inner primer pair (146F2/146R2) to perform the second-step of nested PCR. As in one-step PCR, second step of the nested PCR was also completed, where the reaction mixture was preheated for three minutes at 95°. Thereafter, it was subjected to 35 cycles of one minute denaturation at 95°, one minute annealing at 55°, and one minute elongation or extension at 72°. The last cycle was allowed to finish extending all of the amplified fragments during a final step that lasted five minutes.

### Histopathology

Histopathological process was accomplished according to the handbook of Bell & Lightner [14] for studying penaeid shrimp histology. Briefly, the shrimp specimens were fixed in Davidson Fixative solution and then preserved in 70% ethanol. After fixation, hepatopancraes and muscle tissues from the confirmed WSSV-infected and controlled healthy shrimp samples were dissected carefully and placed inside the cassette. The closed cassette was submerged under 70% alcohol for 16 h or overnight fixation. After that, the cassette was removed from 70% alcohol and was held to automatic tissue processor machine. Dehydration process was conducted by treating the samples with 100% formalin, absolute alcohol and acetone consecutively in separate jars for a specified period of time (four series). The tissue samples were embedded with melted paraffin and then placed on the bottom of a stainless steel mold having perforated plastic holder that facilitated the trimming process. The unmold blocks were placed on the microtome to get paraffin ribbons with a thickness of 5 micrometers. Each of the stretched ribbons was scooped up onto a histological slide and was stained by Hematoxylin and eosin (H & E) stains following a series of treatments. After drying the stained slides, the sections were mounted with DPX mountant. The mounted sections were examined with a compound microscope to produce sharp pictures for documentation and further analysis.

## Results

### Detection and prevalence of WSSV infection in collected shrimp samples

One-step PCR analysis of the 18 samples from the Satkhira district revealed that 15 of the samples were WSSV-infected, while the remaining 3 samples tested negative (Table 2 and Supplementary figures 1–2). However, WSSV infection was discovered in 10 of the Bagerhat district's shrimp samples, while one-step PCR revealed that the remaining 8 samples tested negative for WSSV. Twenty five of the 36 samples were determined to be WSSV positive, as confirmed by one-step PCR. Two-step PCR was used to examine a total of 11 WSSV negative samples that one-step PCR had confirmed WSSV negative. After two steps of PCR, only three samples from Satkhira district were found to be WSSV negative, while eight samples from Bagerhat district tested positive for WSSV (Table 2 and Supplementary figure 3).

**Table 2:** Identification and prevalence of WSSV infection assessed by both one step and nested PCR.

**Sampling areas**	**Farm** no.	**Number of samples**	**One step PCR**		**Two-step nested PCR**			**Prevalence rate (%)** **of WSSV infection**	
			**+(ve)**	**-(ve)**	**Samples those were WSSV negative tested by one step PCR**	**+(ve)**	**-(ve)**	**One step PCR**	T**wo-step nested PCR**
**Satkhira district**	I	6	4	2	3	–	2	83.33	83.33
	II	6	6	–		–	–		
	III	6	5	1		–	1		
**Bagerhat district**	I	6	5	1	8	1	–	55.56	100.00
	II	6	–	6		6	–		
	III	6	5	1		1	–		
**Total**		36	25	11	11	8	3	69.44	91.67

WSSV infection prevalence rates in shrimp samples collected from Satkhira and Bagerhat districts were 83.33% and 55.56%, respectively, according to the one-step PCR results. Thus, overall 69.44% of the shrimp in these small-scale shrimp farms in Bangladesh were found to be WSSV-positive, as confirmed by one-step PCR. Including the two-step nested PCR results, the frequency of WSSV infection raised to 83.33% and 100% in the shrimp samples of Satkhira and Bagerhat districts, respectively (Table 2). Finally, the overall prevalence rate of WSSV infection in the studied shrimp samples was 91.67% determined by combination of both one-step and nested PCR.

### Histopathological studies of healthy and WSSV infected shrimps

**Fig. 1:**
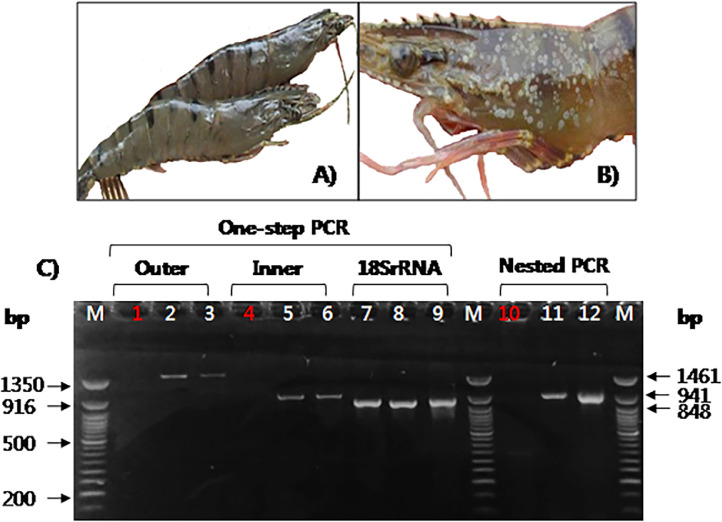
(A-C). Specimens of WSSV non-infected (A) and WSSV-infected (B) *Penaeus monodon* used in histopathogy following confirmation by one-step and nested PCR (C). Lanes 1, 4 & 10 confirmed WSSV negative (healthy control) and lanes 2, 3, 5, 6, 11 & 12 confirmed WSSV positive shrimp samples. Lanes 7, 8 & 9 denoted amplified fragments of shrimp 18SrRNA used as endogenous control to check the quality of the DNA. Lanes M represent 50 bp DNA ladder.

**Fig. 2:**
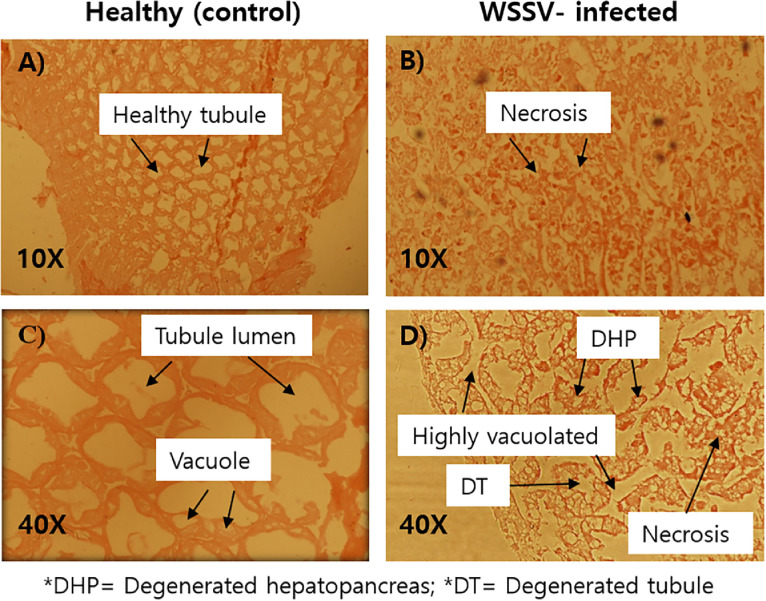
(A-D). Histopathologically stained (H & E) WSSV infected and non-infected hepatopancraeas of *Penaeus monodon*. Histopathological study of HP for non-infected shrimps with 10X and 40X magnifications (A & C) showed healthy tubule (HT), TL (Tubule lumen), and vacuole where no inclusion or necrosis was observed. Infected tissues (B & D) were observed with DHP (Degenerated HP), DT (Degenerated tubule), necrosis, vacuolization of cell at 10X and 40X magnifications.

**Fig. 3:**
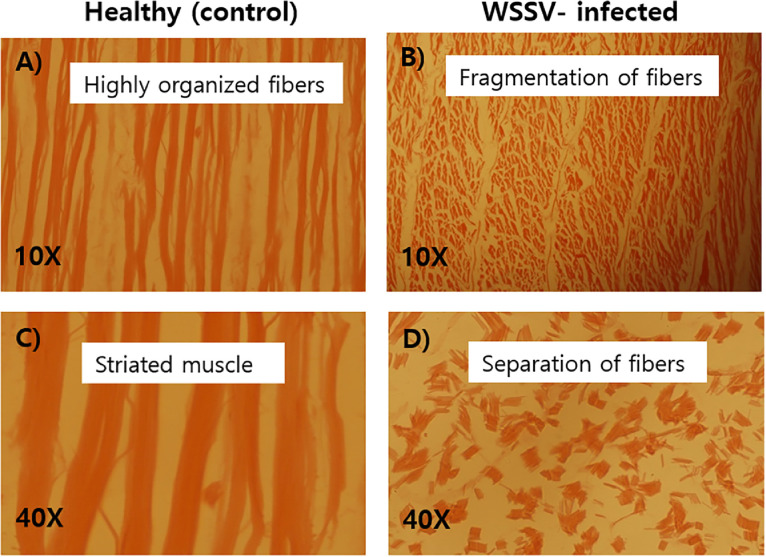
(A-D). Histopathologically stained (H & E) WSSV infected and non-infected muscle of *Penaeus monodon*. Histopathological study of muscle for non-infected shrimps with 10X and 40X magnifications (A & C) showed striated fibers without any damage. Infected tissues (B & D) were observed with loss of myofibrils and muscle striation, fragmentation and separation of muscle fibres at 10X and 40X magnifications.

Prior to histopathological analysis, both WSSV infected and healthy (WSSV negative control) shrimps were confirmed by one-step and two-step nested PCR analysis (Fig. 1). The histopathological study elucidated the destruction or degeneration of hepatopancreatic tissues in the WSSV-infected shrimp as compared to the control healthy shrimp. Histopathology of hepatopancreas (HP) of WSSV infected shrimp was featured by scattered cellular disintegration including degenerated tubule lumen, degenerated and necrotic hepatopancreas (Fig. 2). Degeneration was also observed in epithelial tissue and tubules of hepatopancreas. On the other hand, muscle tissue of WSSV-infected shrimp exhibited the loss of muscle striation, disorganized myofibrils, fragmented and dispersed fibers (Fig. 3).

## Discussion

The most sensitive diagnostic technique for WSSV detection in shrimp is PCR, which outperforms other techniques in this regard [15]. Nested PCR is one of the most commonly used PCR techniques for virus detection including hepatitis C virus [16], bovine herpesvirus [17] and fish rhabdoviruses [18]. In penaeid shrimps, nested PCR was applied to detect WSSV [13], monodonbaculovirus [19], gill-associated and lymphoid virus [20]. In the present study, pleopods and muscle tissue were used. Because the tissues have a larger cell mass than hemolymph, the quantities of WSSV genomes in these tissues were typically much higher than those of hemolymp [21]. To detect WSSV isolates in Taiwan, Lo et al. [13] developed an outer primer pair, 146F1/146R1, and an inner primer pair, 146F2/ 146R2, for one-step and nested PCR, respectively. Ayub and coauthors [10] also reported that both inner and outer primer pairs were useful to amplify the DNA fragments successfully in Bangladeshi WSSV isolates in shrimps. We collected shrimp samples from the marginal shrimp farmers who owned the small scale shrimp farms but usually were not aware much about the viral infection and shrimp diseases. Heavy mortality of shrimps also occurs in their farms due to white spot disease which also contribute to serious loss of the total shrimp industries of the country.

One-step PCR revealed an overall prevalence rate of 69.44% for WSSV-infected shrimp; two-step nested PCR increased this to 91.67%. In Satkhira and Bagerhat districts, the prevalence rates were 83.33% and 100%, respectively. Siddique et al. [22] recorded a WSSV prevalence rate, 78% in the shrimps of Satkhira district. Similar findings were recently reported by Talukder et al. [5], where the Satkhira region had the highest prevalence rate of WSSV infection at 79%, while samples from the Khulna and Bagerhat regions had WSSV prevalence rates of 50% and 38%, respectively. According to Talukder et al. [5], the pre-monsoon season has the highest prevalence of WSSV infection that trending downward during the monsoon season and then rising again in August. According to Mallik et al. [23], infected shrimp in Kolkata, India experienced the lowest pre-monsoon WSSV prevalence and the highest post-monsoon WSSV infection. We investigated the prevalence rates of WSSV infection in cultured shrimps collected during April to May that was typical pre-monsoon period in Bangladesh. We accounted the prevalence rates which were almost similar to other studies [5,22,23].

Only 4% of the PL was positive by one-step PCR, according to Thakur et al. [24], while 49% of the PL was positive by two-step PCR for WSSV. These findings suggested that one-step PCR could identify severely infected samples containing a higher WSSV load, while nested PCR could identify samples with a low level of WSSV infection and WSSV load. However, shrimps showing minor spots or apparently healthy might be positive which could appear as presumed false negative and could not be identifiable by one-step PCR due to fewer amplicons. When viral infection in shrimp was latent or asymptomatic, the chance of being positive by nested PCR was higher than by one-step PCR [25]. Therefore, it is also evident that in nested PCR, the sensitivity of identification and positive results rise as the amplicon size decreases [26].

Histopathology is the diagnostic approach that investigates and examines the tissues and/or cells of diseased or infected specimens after passing through several biochemical and histological processes to investigate the alterations compared with healthy tissues. Hepatopancreas (HP) is a good indicator to identify the shrimp health conditions [27]. The cellular degeneration that was observed in the HP histopathology included widespread degeneration, degenerated tubule lumen (TL), degenerated and sloughing hepatopancreas (SHP) with necrosis, degeneration of the epithelial tissue, collapse of the HP epithelial tubules, and massive sloughing of the epithelial cells in the central HP tubule. Hepatopancreas and gills of shrimps are seriously destroyed owing to WSSV infection. Following one-step and two-step PCR confirmation, our study found histopathological signature of WSSV infection in *P. monodon*. The destruction of HP tissue was observed WSSV-infected shrimp which might be due to WSD. Tubule lumens shrank as a result of the high vacuolation of hepatopancreatic epithelial cells (HECs). The entire tissue of the hepatopancreas of WSSV-infected shrimp was vacuolized, as Wang et al. [28,29] had previously reported. According to Tang and Lightner [21], the sample did not exhibit any symptoms of illness, and the PCR results were only positive in nested reactions, which suggested a low viral load and large Aeosinophilic intranuclear inclusion bodies of the Cowdry type. Our study did not observe that type of inclusion bodies due to later stages of WSSV infection which was even identified by less efficient one-step PCR. Histopathological evidence of necrotic organ, encapsulation, and nodule formation in the hepatopancreas were presented to describe the diagnostic features of the WSSV disease in *P. monodon* [30,31]. Besides, muscle striation and myofibril loss are signs of WSSV infection in abdominal muscle tissue where Zenker's necrosis is very prominent and characteristic observations [32]. In accordance with the observation of Pazir et al. [32], we also observed histopathological signs of in infected abdominal muscle with fragmentation and loss myofibrils and muscle striations.

## Conclusion

Finally, the two-step nested PCR exhibited a sensitive and efficient tool to know more accurate prevalence and detection of WSSV in shrimp and histopathological changes in examined tissues of WSSV infected shrimp would help to understand the mechanism of WSSV infection at tissue level. Further studies should follow immune-histopathological approaches like fluorescent imaging, lateral flow immunoassay (LFIA) to locate binding of polyclonal antibody against WSSV antigen and deploy genetic engineering approaches, i.e., gene editing (CRISPR/Cas systems), silencing of the WSSV responsive receptors and nutrigenomic approaches to improve the antiviral immunity in cultured shrimps against white spot disease and its surveillance for better health care of the highly precious aquaculture species.

## Funding

This work was partially funded by the Sher-e-Bangla Agricultural University Research System (SAURES), Dhaka, Bangladesh.

## CRediT authorship contribution statement

**Md. Juwel Hasan:** Methodology, Data curation, Formal analysis, Writing – original draft. **Shirin Sultana:** Supervision, Methodology, Investigation, Writing – review & editing. **Md. Nasir Khan:** Methodology, Formal analysis. **H.M. Rakibul Islam:** Methodology, Resources. **Mohammad Nazrul Islam:** Conceptualization, Funding acquisition, Project administration, Supervision, Writing – review & editing.

## Declaration of competing interest

The authors declare that they have no known competing financial interests or personal relationships that could have appeared to influence the work reported in this paper.

## Data Availability

Data will be made available on request.

## Appendix Supplementary materials

Image, application 1
