# Supplementary material for: Molecular diagnosis appended by histopathological signature delineates the white spot syndrome virus (WSSV) infection in penaeid shrimps
Source: Comp Immunol Rep. 2024 Feb 13;6:200138. doi: 10.1016/j.cirep.2024.200138 (PMC10884339; doi:10.1016/j.cirep.2024.200138)
Supplement: Supplementary file 1 [file mmc1.doc]

**Supplementary Figures’ Legend**

**Supplementary figure 1**

**
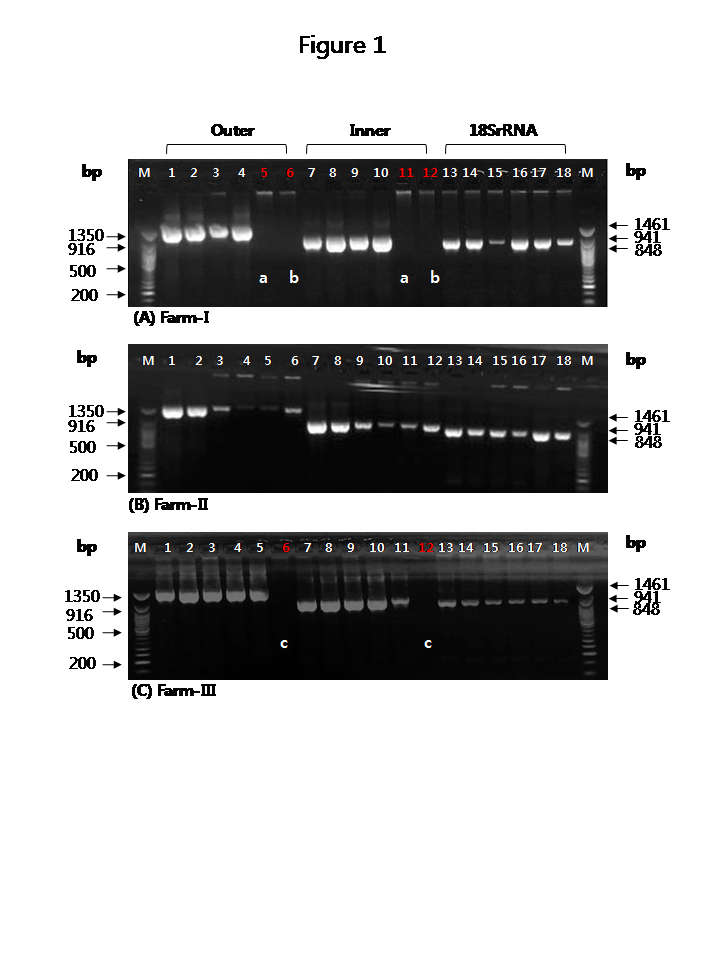
**

Supplementary figure 1 (A-C). Detection of white spot syndrome virus in *Penaeus monodon* collected from three farms of Satkhira district (6 samples/farm) by one-step PCR with outer primers 146F1/146R1 (lanes: 1, 2, 3, 4, 5, 6) and inner primers 146F2/146R2 (lanes: 7, 8, 9, 10, 11, 12). The 143F/145R primer pair was used as endogenous control to check the quality of the DNA (lanes: 13, 14, 15, 16, 17, 18). The letters (a, b and c) under the lane numbers were used for indicating WSSV negative samples. Lanes M represent 50 bp DNA ladder.

**Supplementary figure 2**


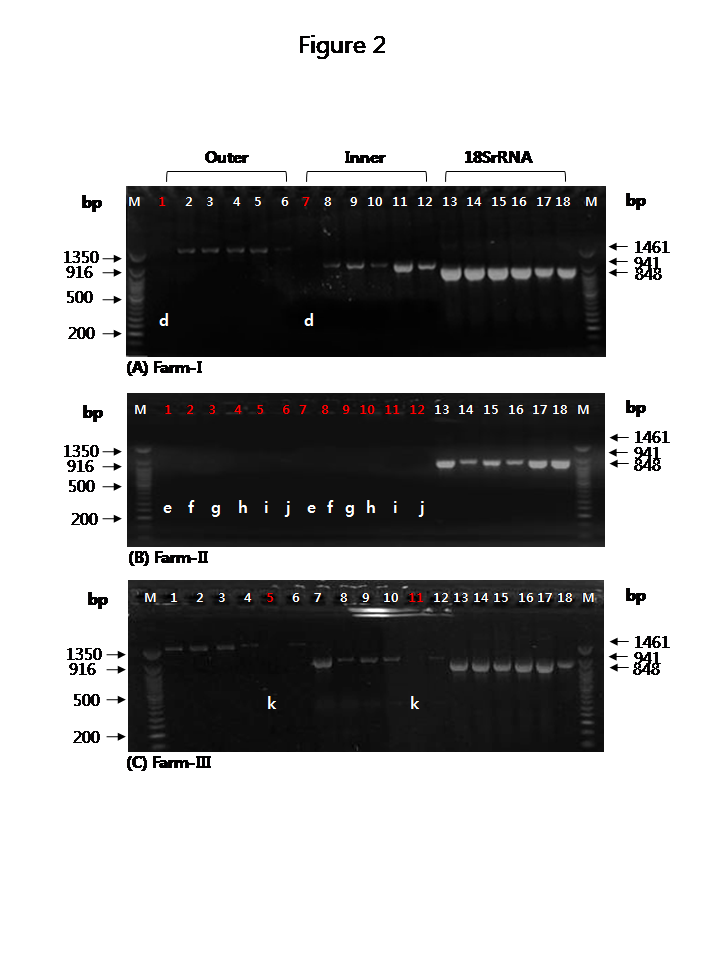


Supplementary figure 2 (A-C). Detection of white spot syndrome virus in *Penaeus monodon* collected from three farms of Bagerhat district (6 samples/farm) by one-step PCR with outer primers 146F1/146R1 (lanes: 1, 2, 3, 4, 5, 6) and inner primers 146F2/146R2 (lanes: 7, 8, 9, 10, 11, 12). The 143F/145R primer pair was used as endogenous control to check the quality of the DNA (lanes: 13, 14, 15, 16, 17, 18). The letters (d-k) under the lane numbers were used for indicating WSSV negative samples. Lanes M represent 50 bp DNA ladder.

**Supplementary figure 3**


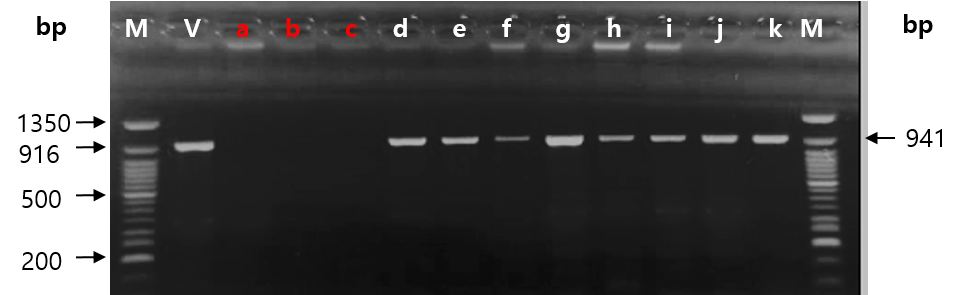


Supplementary figure 3. Detection of white spot syndrome virus (WSSV) in *Penaeus monodon* by two-step nested PCR with inner primers 146F2/146R2 using WSSV negative samples obtained by one-step PCR (lanes: a, b, c, d, e, f, g, h, i, j, k). Lane V denotes WSSV positive control. Lanes M represent 50 bp DNA ladder.
